# Standard to Handheld: A New Wave in Thoracic Ultrasound and Patient Care—A Direct Comparison of Portable Handheld Against Standard in Thoracic Ultrasound

**DOI:** 10.3390/medicina61020313

**Published:** 2025-02-11

**Authors:** Dzufar Halim, Alan Kelly, James Hayes, Kathleen Bennett, Argyrios Tzouvelekis, Dimitrios Ampazis, Fotios Sampsonas

**Affiliations:** 1 Respiratory Department, Cavan General Hospital, H12 A5D7 Cavan, Ireland; dzufarahalim@gmail.com (D.H.); kellyalan@doctors.org.uk (A.K.); jamesphayes@eircom.net (J.H.); dim_ampazis@yahoo.gr (D.A.); 2 Data Science Centre, School of Population Health, Royal College of Surgeons in Ireland (RCSI) Dublin, D02 YN77 Dublin, Ireland; kathleenebennett@rcsi.ie; 3 Respiratory Department, Patras University Hospital, 26504 Patras, Greece; argyris.tzouvelekis@gmail.com

**Keywords:** lung ultrasound, patient care, portable handheld ultrasound device, pleural effusion, interstitial profile

## Abstract

*Background and Objective:* Ultrasound has become more popular and useful over the last few years in improving healthcare. While handheld devices offer portability and convenience, their diagnostic accuracy and clinical utility require further scrutiny. This study attempted to evaluate the non-inferiority of handheld portable ultrasound devices compared to standard ultrasound devices for common lung pathologies. *Materials and Methods:* Videos of various common lung pathologies from 20 patients were recorded by a single operator using both portable handheld and standard ultrasound devices in a single setting. These videos were then assessed via online questionnaires by clinicians of various levels of experience from respiratory and non-respiratory departments. A Likert scale was used, ranging from strongly disagree to strongly agree (ranging from 1 to 5) in terms of overall image quality, clear anatomical visualization, similar clinical interpretations/decisions, and the perception of non-inferiority. Median values with interquartile ranges were reported; a rating of 3 or above was defined as indicating non-inferiority. *Results:* Thirty participants completed the questionnaires, of which the majority were at trainee level (*n* = 20, 73%) and from a respiratory department (*n* = 20, 67%). The participants had mixed levels of experience in terms of the years and frequency of use of the ultrasound. Overall median ratings were 4.0 for overall image quality, clear anatomical visualization, and similar clinical interpretations/decisions, with slight variations in interquartile ranges. No significant differences were observed between subgroups. The portable ultrasound device was rated similarly for the overall perception of non-inferiority, but clinicians from respiratory departments and clinicians with less experience showed statistically significant variability in their assessments. *Conclusions:* The portable handheld device demonstrated potential as a reliable alternative to standard models in standard clinical settings without compromising clinical decision. Further evaluation is needed that includes a direct comparison of various types of handheld ultrasound devices, across different operators’ levels of experience, to further solidify their suitability in patient care.

## 1. Introduction

In recent years, thoracic ultrasound has emerged as a valuable and non-invasive tool in the diagnosis and monitoring of various pulmonary pathologies—including pleural effusions, pneumothorax, pulmonary edema, interstitial lung disease, and pneumonia [1]. Despite their accuracy, well-established standard ultrasound machines are usually limited by their logistical requirements, such as size and costs, which may potentially pose a challenge in resource-limited settings or during the afterhours.

Alternatively, portable handheld ultrasound hardware is being recently advocated in clinical practice as a potential to overcome this logistical barrier. These devices are becoming more advanced in that they are now smaller, battery-operated, and capable of being used in wireless mode, which could facilitate real-time decision making at the bedside. However, their comparability to standard ultrasound devices remains uncertain, particularly with regard to image quality, diagnostic accuracy and overall performance.

While several studies have examined the role of portable handheld ultrasound devices in different settings, few have focused specifically on lung ultrasound. Even fewer studies have directly compared them against standard ultrasound hardware for the lung. The role of handheld devices in thoracic examination has been recognized in international guidelines, but their full adoption as a replacement for standard ones will require further scrutiny, especially to establish their non-inferiority [2].

In Ireland, the Respiratory Integrated Care Service, operating through the Integrated Care Hub in the community, is aimed to improve chronic respiratory care by shifting service from hospital to community settings. These hubs are supported by multidisciplinary teams to deliver early intervention, personalized care plans and preventive measures [3]. However, challenges remain in peripheral areas and outside hospital settings, where access to diagnostic resources for lung examination, is limited. 

Given these challenges, bedside ultrasound presents a promising and effective solution, offering a quicker and timelier approach in both community and hospital settings. The potential integration of handheld devices into both the hospital and community healthcare setting could also enhance continuity of care providing real-time diagnostic capabilities regardless of location. However, a key consideration is whether portable handheld ultrasound devices, which are more cost-effective, could deliver similar findings to standard ultrasound equipment.

## 2. Aim and Objectives

The aim of this study was to evaluate the non-inferiority of handheld portable ultrasound devices compared to standard ultrasound for thoracic examination and common lung pathologies, specifically in terms of overall image quality, clear anatomical structure and similar clinical interpretations or decisions. Additionally, this study attempted to clarify if there were any differences between physicians of different specialities and levels of experience when it comes to evaluating ultrasound images.

## 3. Methods

### 3.1. Study Design

This was a single-centre prospective observational study involving adult patients hospitalized under respiratory teams in Cavan General Hospital, Ireland. Over a 3-month period, videos from 20 patients were recorded and stored. Demographic data of these patients were also collected.

### 3.2. Data Collection

All patients underwent thoracic ultrasound examination with both standard ultrasound (TE5 SP Point of Care Ultrasound Machine) and handheld portable ultrasound (EagleView Wireless Probe Type Ultrasound Scanner) devices by a single operator, in the same single clinical session, at the bedside of the patient in the ward during standard clinical practise. The operator was a respiratory clinician certified by the European Respiratory Society with more than 3 years of experience in ultrasound. No ultrasonographer technician participated in the collection of videos. Lung pathologies were diagnosed by the main operator, primarily based on ultrasound findings. All patients underwent chest X-rays, and some also had CT-thorax scans, either before or after the ultrasound examination. While additional imaging confirmed some of the lung pathologies, the main objective was to compare portable handheld ultrasound with standard ultrasound. The relevant diagnoses were then validated by an independent respiratory physician with over five years of experience in lung ultrasound, who reviewed the images and videos. This respiratory physician was not involved in the care of the patients or the questionnaires. Key findings from the ultrasound were saved on the devices for further comparison at a later stage.

The videos were compiled, de-identified and paired side-by-side (standard and handheld ultrasound) with brief clinical context for each case to be reviewed by a panel of clinicians via an online survey. Clinicians from both respiratory and non-respiratory departments locally and externally were invited to participate.

Participants were asked to evaluate each of the portable handheld ultrasound videos against standard cases using a 5-point Likert scale ranging from 1 (strongly disagree) to 5 (strongly agree), based on the following parameters:

- Overall Image Quality: How clear, detailed, and reliable the images appeared.
- Clear Anatomical Visualization: The extent to which the images clearly displayed relevant anatomical structures.
- Similar Clinical Interpretation or Decision: Whether the images provided enough diagnostic information to support similar clinical decision making between the handheld and standard devices.

Participants were then asked about their perception of the non-inferiority of portable handheld ultrasound devices against standard ultrasound devices using the same scale.

#### Ethical Approval

This project focused on evaluating the image quality of videos obtained from portable ultrasound devices compared to those obtained from standard ultrasound systems. It relied entirely on pre-existing, anonymized videos that were collected and stored in the hospital’s system as part of routine clinical practice and a recent local audit. This audit was approved by the Audit Committee in Cavan Hospital, and registered as audit 202501. Verbal informed consent was obtained from the patients in the audit as part of routine examination and policy. In this project, no patient contact was involved and all videos were anonymized to remove any identifiable patient information, ensuring strict adherence to privacy and confidentiality standards.

As the project involved the secondary use of data, which were collected in accordance with local hospital policies, it aligned with ethical guidelines for audit and service evaluation activities. Furthermore, this study was limited to technical image quality assessment and did not impact patient care or involve any clinical decision making. Given that no new data collection, patient interactions, or identifiable data were involved, this project does not meet the criteria for requiring other ethical approval, beyond the approval of the Audit Committee, remaining compliant with local institutional policies and relevant regulatory standards for data usage.

### 3.3. Statistical Analysis

A 5-point Likert scale, ranging from 1 (strongly disagree) to 5 (strongly agree), was used to collect responses. Given the ordinal nature of these data, descriptive statistics were summarized using median values and interquartile ranges (IQRs). Non-parametric tests were employed for group comparisons using the Mann–Whitney U test for comparisons between two groups and the Kruskal–Wallis test for comparisons involving more than two groups.

For the purpose of this analysis, a Likert scale rating of 3 or above was predefined as indicative of non-inferiority, suggesting that the portable handheld ultrasound performed comparably to the standard device. For the perception of non-inferiority, the responses were divided into two categories: ratings below 3 and ratings of 3 or higher. Fisher’s exact test was used to analyze these categorical data and evaluate significant differences in outcomes across subgroups. A *p*-value of less than 0.05 was considered indicative of statistically significant differences.

## 4. Results

Twenty patients were included, with an average age of 71 years old (range 29–93) and an equal distribution of males and females. These patients had mixed comorbidities. The lung pathologies identified included simple pleural effusion, complex pleural effusion, pneumothorax, interstitial syndrome, consolidation, and atelectasis. Incidental findings of ascites were also noted. These are highlighted in Table 1.

**Figure 1:**
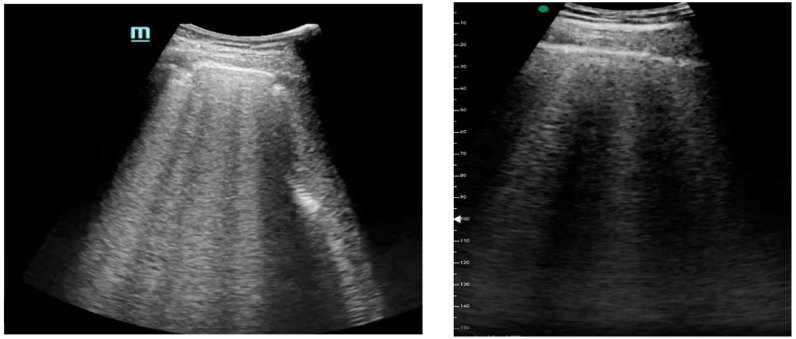
B-lines.

**Figure 2:**
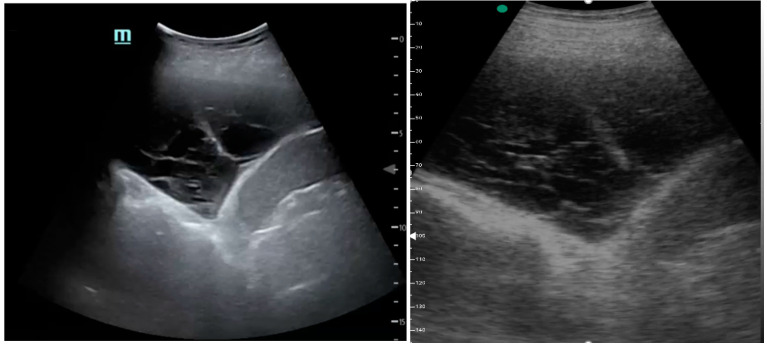
Complex pleural effusion with septation.

**Figure 3:**
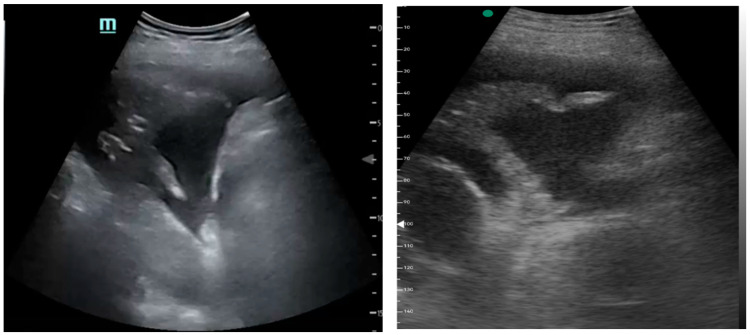
Simple pleural effusion with atelectasis.

Figure 1, Figure 2 and Figure 3 exhibit characteristic examples of ultrasound still images from the videos captured via a standard ultrasound (left side) and portable handheld device (right side). Quality may slightly differ from that of the videos as these are still images.

A total of 30 clinicians participated in this study. Of these, 22 (73%) were doctors in training (trainees) and 20 (67%) were from the respiratory department. Other departments that physicians were working at included intensive care units, emergency, radiology and general medicine departments. The participants varied in terms of their experience with ultrasound, both in terms of the number of years they had used ultrasound devices for and how frequently they used them in clinical practice.

**Figure 4:**
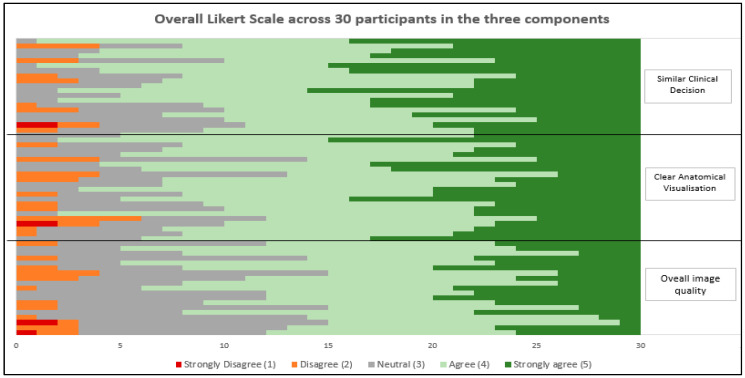
Graphical representation of overall Likert Scale for each participant in the 20 cases.

**Figure 5:**
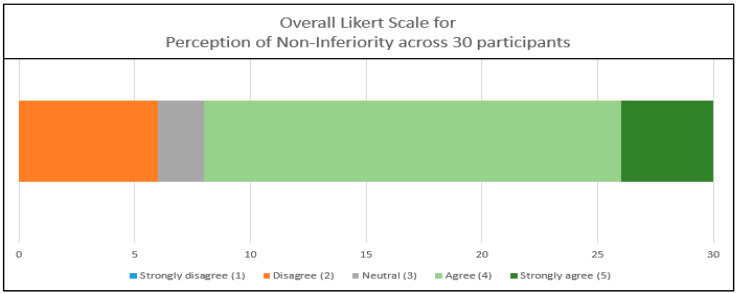
Graphical representation of the overall Likert scale for each participant in all 20 cases for the perception of non-inferiority. The lowest Likert scale value was 2.

The overall Likert scale is illustrated in Figure 4, and the Likert scale for the perception of non-inferiority is illustrated in Figure 5. The median ratings with IQRs across the 30 participants for both the handheld and standard ultrasound devices were as follows:

○ Overall image quality: 4.0 (3–4);
○ Clear anatomical visualization: 4.0 (4–5);
○ Similar clinical interpretations/decisions: 4.0 (4–5);
○ Perception of non-inferiority: 4 (3.25–4).

Table 2 above categorizes the Likert scale into different subgroups. When comparing the subgroups, the overall median rating consistently remained at 4.0, with slight variations in the interquartile ranges across groups. Statistically significant differences were observed in the perception of non-interiority in the subgroups categorized by department (*p* = 0.032) and number of ultrasounds reviewed per year (*p* = 0.026). Clinicians from the respiratory department and those with a lower number of ultrasounds reviewed annually showed greater variability in the interquartile range. Notable variability was also observed specifically in the trainee group when it comes to their non-inferiority rating; however, this difference did not reach statistical significance. Differences in other components were statistically non-significant; see Table 3.

Non-inferiority was defined as a rating of 3 or more on the Likert scale (from Neutral to Strongly Agree). Ratings on the Likert scale for the perception of non-inferiority were recategorized in agreement with those in Table 3. The number of ultrasounds reviewed annually showed a significant association with perceptions of non-inferiority, with those reviewing fewer ultrasounds annually being more likely to rate the device as inferior. A trend toward significance was observed for departmental affiliation (*p* = 0.053), suggesting that respiratory clinicians may perceive the device differently compared to other departments. Other components did not show statistically significant differences; see Table 3.

## 5. Discussion

Across all evaluated criteria—overall image quality, clear anatomical visualization, clinical interpretation, and non-inferiority—the portable handheld ultrasound device consistently performed comparably to the standard ultrasound device. The high median ratings across both ultrasound types suggest that the portable device is viewed as a viable alternative for thoracic examinations for common lung pathologies. This echoes the outcome of the study by Güney (2020), which showed a strong agreement between handheld and standard ultrasound devices in detecting pleural effusion, alveolar syndrome, consolidation and lung aeration [5].

An almost statistically significant difference was observed between physicians working in the respiratory department and those in other departments regarding the perception of non-inferiority, with clinicians from respiratory departments showing more variability in their ratings and clinicians from non-respiratory departments viewing the portable device as being closer in performance to that of the standard device. This could be attributed to frequency of encounters with more complex and challenging clinical cases in respiratory departments compared to other departments. Portable ultrasound devices, while valuable, may be perceived as less reliable in these high-complexity scenarios, potentially explaining the variability in ratings.

A statistical difference was also observed between less and more experienced users. This suggests that more experienced clinicians had more confidence in interpreting images from a portable handheld ultrasound device. The outcomes from the study by Bobia et al. (2018) support this finding as they demonstrated that imaging from a handheld probe is comparable to CT scans when carried out and evaluated by experienced physicians [6]. For training purposes, it is likely more useful for non-consultant doctors or less experienced users to develop a baseline level of competency and confidence with the standard ultrasound before transitioning to the use of portable ultrasound devices, ensuring consistency and reliability in their assessments. However, studies by Graven (2015) and Dalen (2015) indicated that even non-experts such as nurses can be trained in a short period of time to be able to accurately assess pleural effusion and vena cava via portable handheld ultrasound devices, albeit in a predefined situation such as post-cardiac surgery [7,8]. Nevertheless, this study also demonstrates that if users lack confidence in interpreting the findings, images from portable handheld ultrasound devices can be collected and saved for review and discussion with more experienced users at a later time.

Recent studies support the diagnostic equivalence of portable handheld and cart-based ultrasound models. For example, an RCT by Gibbons (2024) found similar diagnostic accuracy between devices, though it included only 11 standard and 7 handheld images [9]. Cricchio et al. (2024) confirmed high accuracy with handheld devices for detecting B-lines, pleural effusions, lung consolidation, and inferior vena cava abnormalities among 72 patients, although the study was limited to just two assessors [10]. Similarly, Jung (2021) evaluated 40 patients with regard to abdominal and pleural conditions and obtained similar outcomes [11]. This study was more focused on abdominal findings and had only two thoracic findings (pleural effusion and pulmonary inflammatory changes). It was also limited to just two assessors.

Our findings align with these studies, showing comparable ratings across all metrics for bedside lung imaging with both standard and portable devices. Our study also involves multiple assessors from different backgrounds and levels of experience in thoracic ultrasound in the context of various common lung pathologies, making it more representative of daily practise scenarios.

## 6. Limitations and Future Directions

Our study has some limitations. This is a single-centre study with a relatively small number of patients, which may have limited the representation of common lung pathologies and relevant findings. A larger sample size with more cases of various lung conditions in a multi-centred study would strengthen the findings and enhance statistical power. 

This study utilized a single operator for both standard and portable handheld ultrasound in a single setting. This approach was chosen to minimize bias and inter-operator variability, allowing the focus to remain on the quality of the video and its clinical relevance. However, relying on a single operator may not fully capture the variability in skills and techniques observed in real-world practice. As a result, the study is limited in its ability to evaluate performance differences across users with varying levels of expertise. Future research incorporating multiple operators would provide a clearer picture of the device’s generalizability and reliability across diverse clinical settings.

Time efficiency is a crucial factor in clinical decision making. This was not assessed in this study. It remains unclear if handheld devices require more time for assessment compared to standard devices. A previous study by Dewar et al. (2020) indicated that no significant difference was observed in image acquisition time between handheld and standard ultrasound [12]. However, the study was conducted exclusively on a healthy population. More recently, a study by Acuna (2024) demonstrated that handheld device had significantly faster FAST (Focused Assessment with Sonography for Trauma) examination scores in 62 patients compared to standard ultrasound [13].

The image quality of handheld devices may vary depending on the brands and manufacturer, which can affect usability, image quality and diagnostic accuracy. Perez-Sanchez (2024) evaluated multiple handheld devices from major ultrasound brands with different assessors [14]. These were not, however, tested against standard ultrasound devices. In our study, only one brand of handheld devices was compared against a standard device to reduce variability in image quality due to different equipment. This approach allowed for a focused assessment of the device performance. Expanding future research to include multiple devices from various manufacturers would provide a more comprehensive evaluation of handheld device capabilities against those of standard ultrasound.

The devices used in this study were not from the same brand, which could limit comparability between the portable and standard devices. However, this reflects a real-world scenario, particularly in resource-limited settings where logistical barriers often prevent the use of matching brands. Despite this limitation, our study demonstrated that even with different brands, diagnoses were obtainable and accuracy remained consistent.

An equal number of clinicians in each group were offered to participate in the questionnaires. However, the response rate was asymmetrical, as only a certain number responded within the given timeframe. Most respondents were trainees and from the respiratory department, leading to an imbalance that may limit the generalizability of the findings to broader clinical teams. Nonetheless, our findings provide a valuable insight into training needs, an area that, to the best of our knowledge, has not been addressed. This study suggests that handheld ultrasound devices may be most reliable and safe when used by clinicians who review at least 50 ultrasounds annually.

Additionally, clinicians were categorized based on their years of experience and frequency of ultrasound use. However, their prior experience with portable handheld ultrasound devices specifically was not clarified. This omission may have introduced bias in some evaluations. Future studies should address this limitation to provide a more robust evaluation of handheld devices.

## 7. Conclusions

This study demonstrates that portable handheld ultrasound devices have the potential to serve as a viable alternative to standard ultrasound systems for thoracic examinations. Across key metrics—image quality, anatomical visualization, and clinical interpretation—handheld ultrasound devices consistently performed comparably to standard ultrasound devices, offering similar diagnostic accuracy. While this study has limitations, handheld ultrasound was generally perceived as non-inferior. Further research involving direct comparisons of various handheld devices across operators with different levels of experience will provide valuable insights into their broader applicability and potential to enhance patient care.

## Figures and Tables

**Table 1:** Patients’ demographic characteristics including lung pathologies identified from the ultrasound examination.

Patient Characteristics	
Age, years	71 (28–93)
Gender, M/F	9/11
Comorbidities	
Type 2 Diabetes Mellitus	5 (25%)
Hypertension	8 (40%)
Ischaemic Heart Disease	5 (20%)
Congestive Cardiac Failure	5 (20%)
Atrial Fibrillation	5 (20%)
COPD	3 (15%)
Chronic Kidney Disease	2 (10%)
Dementia	3 (15%)
History of cancer (current or previous)	9 (45%)
Lung pathologies	
Simple pleural effusion *	10 (50%)
Complex pleural effusion **	
Echogenic effusion without septation	5 (25%)
Echogenic effusion with septation	5 (25%)
Pneumothorax	1 (5%)
Interstitial syndrome/B-lines	4 (20%)
Consolidation/Air bronchogram	5 (25%)
Atelectasis	9 (45%)
Incidental findings (ascites)	2 (10%)

* Simple pleural effusion is an anechoic effusion which appears black on ultrasound imaging. ** Complex pleural effusion is categorized into echogenic pleural effusion with or without septation. When a pleural effusion is echogenic, it contains bright cellular debris or particles swirling within it [4].

**Table 2:** This table summarizes the findings and subgroup analysis from comparisons made across each group.

Demographic	Frequency, *n* (%)	Overall Image Quality, Median (IQR)	Clear Anatomical Visualization, Median (IQR)	Similar Clinical Interpretation or Decision, Median (IQR)	Perception of Non Inferiority, Median (IQR)
Roles					
Consultants Trainees	8 (27%)	4.0 (3.75–4.0)	4.0 (3.88–4.25)	4.0 (4.0–4.13)	4.0 (4.0–4.25)
	22 (73%)	4.0 (3.5–4.0)	4.0 (4.0–4.38)	4.0 (4.0–5.0)	4.0 (2.25–4.0)
Departments					
Respiratory Others	20 (67%)	4.0 (3.38–4.0)	4.0 (3.88–4.0)	4.0 (4.0–5.0)	4.0 (2.0–4.0)
	10 (33%)	4.0 (4.0–4.0)	4.0 (4.0–4.88)	4.0 (4.0–4.50)	4.0 (4.0–4.75) *
Experience with ultrasound					
<3 years	15 (50%)	4.0 (3.5–4.0)	4.0 (3.75–4.25)	4.0 (4.0–5.0)	4.0 (2.5–4.0)
>3 years	15 (50%)	4.0 (4.0–4.0)	4.0 (4.0–4.5)	4.0 (4.0–4.25)	4.0 (4.0–4.0)
No. of ultrasounds reviewed per year					
10–50	12 (40%)	4.0 (3.75–4.25)	4.0 (4.0–4.5)	4.0 (4.0–4.13)	3.5 (2.0–4.0)
50–100	9 (30%)	4.0 (3.5–4.0)	4.0 (4.0–4.0)	5.0 (4.0–5.0)	4.0 (4.0–4.0)
>100	9 (30%)	4.0 (4.0–4.0)	4.0 (4.0–4.0)	4.0 (4.0–4.0)	4.0 (4.0–4.0) **

* *p* < 0.05 for comparison between groups (non-parametric Mann–Whitney U test, *p* = 0.032). ** *p* < 0.05 for comparison between groups (non-parametric Kruskal–Wallis test, *p* = 0.026).

**Table 3:** This table summarizes the association between the various demographic and professional characteristics of the physicians who participated in the study and the perception of non-inferiority (<3 versus ≥3 on the Likert scale).

	Perception of Non-Inferiority		*p* Value
	Likert Scale < 3	Likert Scale 3+	
**Role**			*p* = 0.099
Consultant	0	8	
Trainees	6	16	
**Department**			*p* = 0.053
Respiratory	6	14	
Others	0	10	
**Years of experience**			*p* = 0.361
0–3 years	4	11	
3+ years	2	13	
**Number of US reviewed annually**			*p* = 0.045 *
10–50	5	7	
50–100	1	8	
100+	0	9	

* *p* < 0.05 for comparison between groups (Fisher’s exact test).

## Data Availability

Data is available to the Audit and Quality Improvement Committee of the institution and can be provided to the editor upon request.
